# MicroRNA-21 Silencing in Diabetic Nephropathy: Insights on Therapeutic Strategies

**DOI:** 10.3390/biomedicines11092583

**Published:** 2023-09-20

**Authors:** Yogita Dhas, Numair Arshad, Nupur Biswas, Lawrence D. Jones, Shashaanka Ashili

**Affiliations:** 1 Rhenix Lifesciences, Hyderabad 500038, India; 2 CureScience, 5820 Oberlin Dr 202, San Diego, CA 92121, USA

**Keywords:** nephropathy, chronic kidney disease, diabetes, pharmacological silencing of miR-21, LNA-21, antagomirs, small interfering RNAs (siRNAs), antisense oligonucleotides (ASOs), miR-21-5p, miR-21-3p, hsa-miR-21

## Abstract

In diabetes, possibly the most significant site of microvascular damage is the kidney. Due to diabetes and/or other co-morbidities, such as hypertension and age-related nephron loss, a significant number of people with diabetes suffer from kidney diseases. Improved diabetic care can reduce the prevalence of diabetic nephropathy (DN); however, innovative treatment approaches are still required. MicroRNA-21 (miR-21) is one of the most studied multipotent microRNAs (miRNAs), and it has been linked to renal fibrosis and exhibits significantly altered expression in DN. Targeting miR-21 offers an advantage in DN. Currently, miR-21 is being pharmacologically silenced through various methods, all of which are in early development. In this review, we summarize the role of miR-21 in the molecular pathogenesis of DN and several therapeutic strategies to use miR-21 as a therapeutic target in DN. The existing experimental interventions offer a way to rectify the lower miRNA levels as well as to reduce the higher levels. Synthetic miRNAs also referred to as miR-mimics, can compensate for abnormally low miRNA levels. Furthermore, strategies like oligonucleotides can be used to alter the miRNA levels. It is reasonable to target miR-21 for improved results because it directly contributes to the pathological processes of kidney diseases, including DN.

## 1. Introduction

Diabetes mellitus is a multifactorial and chronic metabolic disorder characterized by hyperglycemia and caused by defects in insulin secretion or action. In both developed and developing countries, diabetes is a major public health concern. In the last 20 years, the global diabetes population has more than doubled [1,2,3,4,5] and type 2 diabetes mellitus (T2DM) accounts for more than 90% of the global diabetes burden [5,6,7]. In diabetic patients, the long-term and recurring hyperglycemic condition causes dysfunction, internal organ injury, and failure, which leads to the development of diabetes-related comorbidities [8].

Among all diabetes-related comorbidities, diabetic nephropathy (DN) is regarded as one of the leading causes of end-stage renal disease (ESRD), which further leads to increased morbidity and mortality in diabetic patients [9]. DN imposes the highest burden in terms of both financial expense and impact on daily life [10,11]. In the 1980s, Mogensen defined diabetic kidney disease (DKD) as a progressive condition that is initiated with the loss of small amounts of albumin into the urine (30–300 mg/day), termed microalbuminuria or incipient nephropathy [12]. DN has generally been considered a microvascular ailment, with retinopathy [13] and neuropathy [14], and is distinct from macrovascular disease, which leads to peripheral vascular disease, coronary heart disease, and cerebrovascular disease [15]. However, each condition can be considered as a tissue-specific expression of the same pathogenetic process, with DN being the renal manifestation of the same glucose-driven process that occurs at other vulnerable places in the body [16,17,18].

On the other hand, in the past decades, the dysregulation of small non-coding RNA molecules, and miRNAs sparked interest in the occurrence and progression of DN. Several microRNAs (miRNAs) have been reported to be deregulated in DN, including miR-21, miR-124, miR-135a, miR-192, miR-195, miR-200, miR-215, miR-216a, miR-217, miR-377, and miR-1207-5p, which bind to the 3′ untranslated region (UTR) of reno-protective genes and suppress their activity [19]. Thus, miRNAs can serve as a diagnostic tool and be used to anticipate the involvement of certain pathogenic signaling in DN [8,10,20]. Among the miRNAs, miR-21 is one of the most significant miRNAs involved in renal fibrosis and is elevated in the tissues of the kidneys [21]. Overexpressed miR-21 increases oxidative stress and regulates signaling pathways related to renal fibrosis. In mice, miR-21 promotes renal fibrosis by silencing metabolic pathways [22]. It is a highly regulated miRNA in kidneys of the mouse model for DN [23]. In this context, the strategic use of miR-21 as a therapeutic target may be helpful to combat DN.

In this narrative review, we explored the current understanding of miR-21 in the development and pathogenesis of DN. We addressed its association with metabolic pathways and discussed the possible strategies to use miR-21 as a therapeutic agent. Finally, we discussed the future possibilities and concerns related to miRNA-based therapy.

## 2. Development of DN and Current Therapeutics

The inability of endothelial cells to downregulate their glucose transport in response to high glucose levels, in particular, results in an overwhelming flux of intracellular glucose, triggering the production of pathogenetic mediators that contribute to the development of DKD [11]. Pathogenetic processes, initiated and sustained in the kidney by increased glucose levels, can be intensified by various variables. These include a range of metabolic (glucose-dependent pathways) alterations, hemodynamic (various vasoactive hormones) abnormalities, and alterations in signaling pathways caused by prolonged hyperglycemia resulting in the development of DN. The key factors in the development and progression of renal dysfunction are almost always present, such as excess fatty acids, oxidative stress, dyslipidemia, obesity, hypertension, formation of advanced glycation end products (AGEs), the activation of protein kinase C (PKC), inflammation, intrarenal vascular disease, acute kidney injury, glomerular atherosclerosis, activation of the renin–angiotensin–aldosterone system (RAAS), and age-related nephron loss. In the context of diabetes, these factors feed into and intensify common pathogenetic pathways such as elevated levels of growth factors, vasoactive hormones, cytokines, and chemokines in the kidney [11,24].

Current treatments for DN include drugs to slow its progression or kidney replacement therapy, neither of which is an effective treatment for DN. The current standard of care for DN involves blood pressure control with angiotensin I-converting enzyme inhibitors or angiotensin receptor blockers and good glycemic control, which have been partially effective in delaying the onset and/or progression of renal dysfunction. Additionally, metformin combined with sodium-glucose co-transporter 2 inhibitors (SGLT2is) is recommended as the first-line treatment for patients with a glomerular filtration rate ≥ 30 mL/min/1.73 m^2^ because of their cardioprotective properties and ability to stop the progression of chronic kidney disease (CKD). Concomitant mineralocorticoid receptor blockade has been employed to further prevent the CKD progression. A combined regimen of angiotensin I-converting enzyme inhibitors or angiotensin receptor blockers with a mineralocorticoid receptor blockade may help to further reduce albuminuria in diabetic nephropathy [24].

The best practices to address this devastating condition are rapid diagnosis and proper interventions. Early identification has the potential to offer long-term advantages by decreasing disease progression, increasing life expectancy, and minimizing the humanistic and economic burden. Despite these advantages, DN instances are rarely discovered until serious issues arise [11,24].

A better knowledge of the molecular pathways that are dysregulated in the early stages and progression of the disease is critical for the development of novel therapies for this catastrophic condition. Identifying early biomarkers will support the discovery of new mechanisms of pathophysiological alterations in diabetic kidney injury. Although numerous molecular mechanisms have been addressed as therapeutic approaches in DN, the incidence and prevalence of the disease are increasing. On these grounds, identifying a novel target(s) for a new therapy to improve the clinical management of DN is vital [11,24,25].

## 3. MicroRNAs in Diabetic Nephropathy

miRNAs are endogenous, non-coding RNA molecules that are 19–25 nucleotides long [20]. These small molecules attach to the 3′ UTR of target messenger RNAs (mRNAs) [26]. miRNAs regulate the gene expression of particular mRNA targets and are produced in the cell nucleus via a complicated multi-step biosynthetic process that begins with RNA polymerase II. The human genome harbors approximately more than 2300 miRNAs [27], with each possessing the potential to regulate the activity of hundreds of mRNAs. These miRNAs are recognized for their capability to modulate the expression of over 60% of protein-encoding mRNAs, exhibiting tissue and cell-specific regulatory effects. They can be found in plasma, urine, cerebrospinal fluid, and other extracellular fluids. miRNAs are involved in a variety of cellular activities, including differentiation, cell growth and proliferation, metabolism, organogenesis, tissue remodeling, stress response, and apoptosis. Furthermore, they have important roles in many diseases, including neurological disorders, cancer, vascular disease, heart disease, viral infection, diabetes mellitus, and diabetes-related kidney disease [10,20,28].

miRNAs play a pivotal role in various aspects of DN pathophysiology, encompassing the pathological alterations of the glomerular basement membrane, the buildup of extracellular matrix proteins such as collagen and fibronectin, and the process of epithelial-to-mesenchymal transition (EMT). These aforementioned changes collectively contribute to the characteristic manifestation of renal tissue fibrosis [9].

**Figure 1:**
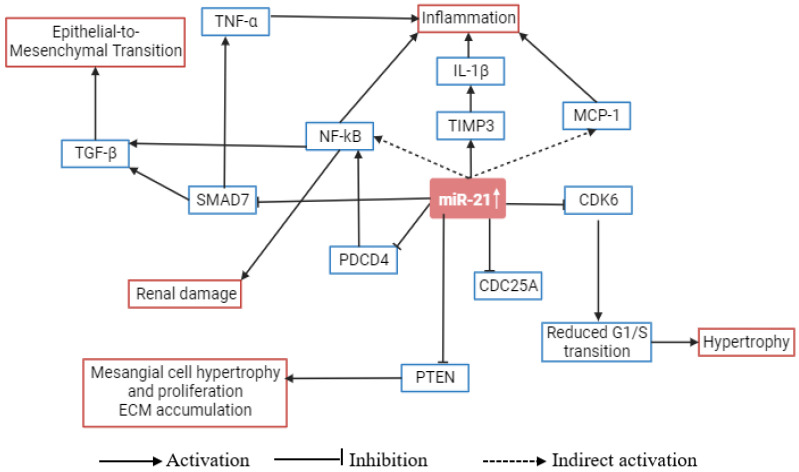
Role of miR-21 in the pathogenesis of diabetic nephropathy. miR-21, microRNA-21; PTEN, phosphatase and TENsin homolog; TGF-β, transforming growth factor-β; TIMP3, tissue inhibitor of metalloproteinases 3; MCP-1, monocyte chemoattractant protein 1; CDC25A, cell division cycle 25A; CDK6, cyclin dependent kinase 6; NF-κB, nuclear factor κB; PDCD4, programmed cell death 4; IL-1β, interleukin-1β; TNF-α, tumor necrosis factor α [10,23].

One of these miRNAs, miR-21, has been one of the most studied multipotent miRNAs. Its expression undergoes significant alterations in DN, highlighting its relevance in the context of renal fibrosis. Figure 1 shows the role of miR-21 in the pathogenesis of DN. miR-21 is often investigated to enhance cell proliferation, inflammation, angiogenesis, and immunological destruction and has been implicated in the development of DN in numerous studies. Also, prior studies have reported that overexpression of miR-21 under high glucose settings suppresses mesangial cell proliferation, and while exaggerating Akt activation stimulates mesangial cell hypertrophy and fibronectin expression [29,30]. The degree of glomerular fibrosis during renal fibrosis has been strongly correlated with miR-21 expression levels. miR-21 overexpression increases transforming growth factor-beta1 (TGF-β1)-induced EMT by upregulating mothers against decapentaplegic homolog 3 (SMAD3) expression and downregulating SMAD7 expression. Notably, miR-21 inhibitors improve kidney structure and function in DN in addition to halting the advancement of renal fibrosis and EMT [31,32]. Thus, the direct reduction of renal fibrosis in DN may be achieved by suppressing miR-21.

## 4. Role of microRNA-21 in Metabolic Pathways Related to Diabetic Nephropathy

miR-21 is classified as a non-coding miRNA, with a length ranging from 20 to 24 nucleotides and is encoded by gene MIR21. Its chromosomal location is 17q23.1 [33]. The main function of miR-21 is the degradation of mRNAs of several target genes by binding to their 3′ UTR regions [34]. The biogenesis of miR-21 begins with the expression of primary transcript pri-miR-21. The mature miR-21 is formed by two consecutive cleavage reactions [35]. The gene coding pri-miR-21 overlaps with the TMEM49 gene.

miR-21 has several experimentally validated target genes including programmed cell death 4 (PDCD4), retinoblastoma 1 (RB1), transforming growth factor beta receptor 2 (TGFBR2), B-cell lymphoma-2 (BCL-2), phosphatase and TENsin homolog (PTEN), SMAD7, tropomyosin 1 (TPM1), tumor protein P63 (TP63), and others (Figure 1). Many of them are linked to intrinsic or extrinsic pathways of apoptosis and autophagy. As it is overexpressed in many cancers, it is considered an oncogenic miRNA. Its oncogenic function includes inhibition of the apoptosis pathway [36,37]. In the case of pancreatic cancer, miR-21 promotes glycolysis and lactate production. This metabolic reprogramming favors cancer progression [38]. For renal cancer, it promotes malignancy by silencing the large tumor suppressor gene 1 (LATS1) [39].

In the case of DN also, miR-21 is upregulated and its target genes have critical roles in multiple signaling pathways which regulate renal fibrosis [40]. DN is associated with progressive renal fibrosis. Fibrosis originates from tissue injuries and is caused by the activation of renal fibroblasts which secrete and remodel the extracellular matrix [41]. The extracellular matrix proteins accumulate in the mesangium and basement membrane of the glomerulus and renal tubulointerstitium. The changes in the protein composition in the mesangium are initiated by glucose metabolism and the formation of advanced glycation end products [42]. Overexpression of glucose transporter type 1 (GLUT1) in mesangial cells enhances the synthesis of an extracellular matrix [43,44]. As a consequence of glucose metabolism, reactive oxygen species (ROS) are generated which further activate several intracellular signaling pathways [45]. The signaling pathways activate redox-sensitive transcription factors which change gene expressions of extracellular matrix proteins [42]. In normal injury, fibrosis helps to repair damaged tissues. In pathological fibrosis, the activation of fibroblasts is prolonged [46]. miR-21 levels were found related to increased fibrosis through the TGF-β signaling pathway. Wang et al. reported knockdown of Ski-related novel protein (SnoN) which promotes the upregulation of miR-21 [47]. SnoN is known to negatively regulate the TGF-β signaling pathway which promotes the synthesis of extracellular matrix proteins [48,49]. Hence, SnoN suppresses renal fibrosis by modulating the TGF-β signaling pathway and miR-21 expression [47].

On the other hand, it was also reported that the overexpressed miR-21 can promote fibrosis by silencing metabolic pathways in mice [22,50]. Chau et al. reported that in miR-21 knockdown mice, 700 genes belonging to metabolic pathways are de-repressed. The pathways included fatty acid and lipid oxidation pathways [50]. Alternatively, in murine models, the overexpression of miR-21 has the capacity to suppress metabolic pathways. Roggli et al. reported that the overexpressed miR-21 reduces glucose-stimulated insulin secretion [51]. In addition, Liu et al. observed that in mice lacking miR-21 specifically in β-cells, there was an enhancement in glucose uptake and an increase in glucose-stimulated insulin secretion [52]. Additionally, it has been revealed that miR-21 contributes to cardiovascular diseases. miR-21, which was initially shown to promote tumor growth, was later determined to be involved in mediating the homeostasis of the cardiovascular system. Abnormally high levels of miR-21 cause a wide range of cardiovascular diseases, such as coronary heart disease, cardiac fibrosis, and cardiac hypertrophy. In turn, miR-21 increases heart hypertrophy and interstitial fibrosis by encouraging cardiac fibroblast survival and growth factor release [53].

Furthermore, it has been observed that the overexpression of miR-21 is related to the prognosis of DN through renal fibrosis. However, this relationship appears to be predominantly influenced by signaling pathways rather than metabolic pathways. Further investigations are warranted to explore the specific impact of upregulated miR-21 on genes involved in metabolic pathways.

## 5. Strategies to Use miR-21 as a Therapeutic Target in Diabetic Nephropathy

**Figure 2:**
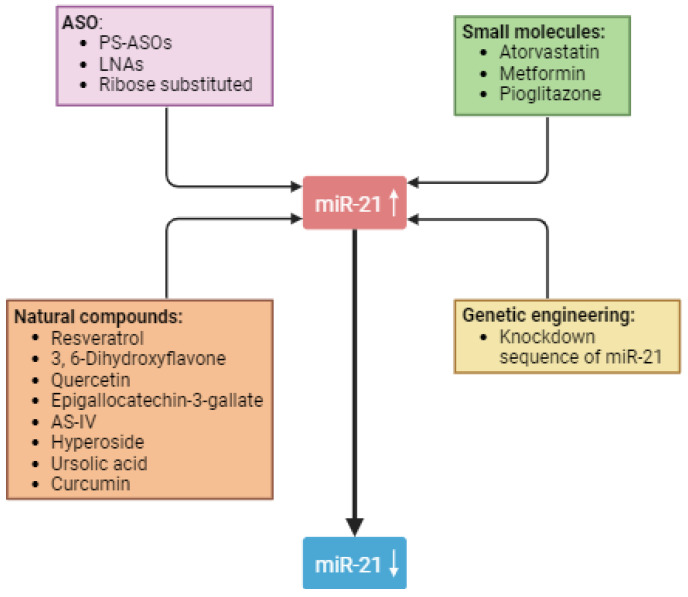
Strategies to use miR-21 as a therapeutic target. Abbreviations: AS-IV: astragaloside IV; ASO: antisense oligonucleotide; LNA: locked nucleic acid.

Currently, available experimental interventions offer a way to rectify the lower miRNA levels as well as to reduce excessive levels. Figure 2 shows possible strategies for lowering miR-21 levels. The use of synthetic miRNAs, commonly known as miR-mimics, can compensate for the lower-than-usual levels of miRNAs. In addition, strategies such as oligonucleotides can be employed to reduce the levels of miRNAs. Since miR-21 plays a direct role in the pathological processes of various CKDs, including DN, it is rational to target it for better outcomes. Targeting miR-21 offers an advantage in terms of selectivity in DN as opposed to transcription factors, which are complex molecules and present a pharmacological challenge in specific targeting. Currently, miR-21 is being pharmacologically silenced through various methods, all of which are in early development. In Table 1 we summarized different targets of miR-21 and their therapeutic implications in DN.

### 5.1. Antisense Oligonucleotides

Oligonucleotides are short, synthetic sequences of nucleotides, either DNA or RNA, that find their uses in genetic applications from DNA sequencing to modulation of gene expression. Antisense oligonucleotides (ASOs) are a type of oligonucleotide that can be designed to bind and inhibit the function of an RNA such as miR-21 [59]. ASOs have advanced enough to provide precise targeting of miR-21, making it a highly feasible strategy; however, they have a major drawback that has limited their use in in vivo applications. They are extremely sensitive and vulnerable to ribonuclease-mediated degradation, which occurs in both cellular and extracellular environments. When injected into the kidneys of a diabetic animal, they are degraded by ribonucleases in the bloodstream. To circumvent this limitation of ASOs, the nucleotides are chemically modified to prevent them from ribonuclease-mediated degradation and to be quickly taken in by the target cells. The results of these modifications have produced 3 types of oligonucleotides. (1) The least modified ASO is obtained by substituting non-bridged phosphate oxygens with sulfur atoms. The resulting species are called phosphorothioate (PS)-modified ASOs which are resistant to ribonucleases and stay intact for a longer period. However, they lack specificity in terms of binding to their targets and their unwanted involvement with the proteins presents another significant drawback [60]. (2) A methyl bridge is inserted between 2-O and 4-C of the ASOs to form locked nucleic acids (LNAs). This modification gives LNAs a locked 3′-endo conformation which is protective against nucleases. A modification known as GapmeR has been employed to improve the function of miRNAs [61]. This modification involves connecting two LNAs with a short DNA segment. As a result, the modified species, known as GapmeR, exhibits increased susceptibility to degradation by nucleases when bound to their targets. This enhanced degradation is considered a desirable property in the context of their intended function [61]. (3) The third and most advanced type of ASO is obtained through the substitution of ribose 2′-OH group with 2′-O-methyl (2′-O-Me), 2′-fluoro (2′-F), or 2′-O-methoxyethyl (2′-O-MOE). It is also resistant to nucleases and has additional advantages such as increased half-life, higher specificity, and being non-immunogenic [62]. Other types of ASOs can also be formed through a mix and match of these modifications to meet the desired properties. One such example is antagomir which contains 2′-O-Me, PS, and a cholesterol group [63].

Various types of ASOs have been used in animal studies to evaluate the therapeutic effects of miR-21 silencing. In one such preclinical study, the researchers first established that miR-21 is a major unregulated miRNA in DN animal models and human patients [23]. They also found that miR-21 suppresses the cell division cycle 25A (CDC25A) and cyclin-dependent kinase 6 (CDK6) in mesangial cells that promote a G1-phase arrest, eventually leading to hypertrophy (Table 1). Moreover, a higher miR-21 level could induce podocyte motility by regulating PTEN [30]. Subsequently, they proceeded to suppress miR-21 in diabetic mice by employing LNAs, leading to a decrease in tubulointerstitial fibrosis, mesangial matrix expansion, and albuminuria [23]. A single dose of LNAs showed its effects for at least 21 days indicating the sustainability of this mode of treatment.

Exploring beyond direct DN, work has been done on the therapeutic silencing of miR-21 in Alport syndrome which shares many features with DN. In the context of this article, the most important features present between Alport syndrome and DN are miR-21-induced glomerulosclerosis and tubulointerstitial fibrosis. In a preclinical study published in 2015, the researchers performed miR-21 silencing in a mouse model of Alport nephropathy [22]. The mice were subcutaneously administered ASOs that were chemically modified to enhance their stability, specificity, and compound visualization in the kidneys. This intervention led to a lowering of albuminuria and attenuation of glomerulosclerosis and interstitial fibrosis. In another animal study that evaluated the individual and combined effects of anti-miR-21 ASOs (Lademirsen) with angiotensin I-converting enzyme inhibitor therapy (ramipril) in Alport syndrome mice models; anti-miR-21 therapy was found to have both individual and additive benefits in delaying kidney damage [64]. Lademirsen also underwent a phase 1 clinical trial for its safety and pharmacodynamic and pharmacokinetic profile in Alport syndrome patients but its phase 2 clinical trial was terminated by the sponsor because it failed to meet the pre-defined futility criteria [65].

### 5.2. Natural Compounds

Oligonucleotides are not the sole method to down-regulate miR-21; some natural compounds such as resveratrol, 3, 6-dihydroxyflavone, quercetin, and epigallocatechin-3-gallate, can also lower the levels of miR-21 [66]. Astragaloside IV (AS-IV), a bioactive saponin extracted from the Astragalus root, is another such compound that downregulates miR-21. In a study conducted in 2017, the authors investigated the effect of AS-IV on the progression of DN in diabetic mice and high glucose-treated podocytes [67]. They found that AS-IV treatment, in a dose-dependent manner, significantly ameliorated progressive albuminuria and glomerulosclerosis in diabetic mice. AS-IV also attenuated high glucose-induced podocyte apoptosis, caused remission in the endoplasmic reticulum (ER) stress, and restored impaired autophagy. Additional investigations looking into the underlying mechanism of these effects revealed that the protective effects of AS-IV were mediated, at least in part, by sarcoplasmic/endoplasmic reticulum Ca^2+^ ATPase (SERCA2)-dependent ER stress attenuation and activation of protein kinase α (AMPKα)-promoted autophagy induction pathways. Another study found that the miR-21-inhibiting ability of AS-IV improved renal function and renal fibrosis [68]. The detailed analysis showed that overexpression of miR-21 activated the β-catenin pathway and the TGF-β1/Smads pathway which in turn promoted podocyte dedifferentiation and mesangial cell activation. The downregulation of miR-21 reversed these processes and led to an improvement of renal function in the mice models.

Hyperoside is a natural compound obtained from Abelmoschus manihot L medic and is known to have a hypoglycemic effect. In a study by Zhang et al., the administration of hyperoside markedly improved the renal dysfunction of diabetic mice [69]. These effects of hyperoside were mediated by suppression of fibronectin (FN), collagen IV (COL IV), and TIMP1 expression, and promotion of matrix metalloproteinase-9 (MMP-9) and MMP-2 expressions. The up-regulation of MMP-9 in a post-transcriptional manner led the authors to speculate that hyperoside shows its effects by downregulating miR-21. Additional analysis revealed that hyperoside exerts a down-regulatory effect on miR-21 expression.

Ursolic acid is known to have anti-hyperglycemic, anti-hyperlipidemic, anti-inflammatory, and anti-oxidative effects. Previously, it was shown to inhibit miR-21 and cause apoptosis and ameliorate fibrosis and hypertrophy [70,71]. In a study evaluating the effects of ursolic acid in DN, it was shown to ameliorate podocyte injury in high glucose-treated murine cell cultures. These effects were secondary to the inhibition of miR-21 which increased the expression of PTEN which in turn inhibited Akt and mTOR and restored autophagy and eventually ameliorated renal injury [72].

Quercetin is another plant-based compound known to have ant-miR-21 effects. It has been shown to ameliorate diabetic fibrosis in human tubular epithelial HK-2 cells by downregulating miR-21 [73]. A lack of miR-21 upregulated PTEN and TIMP3 which were already known to play an anti-fibrotic role in the kidneys [74,75].

Curcumin also possesses anti-miR-21 activity. However, it has limited bioavailability. Very high doses are required to achieve its therapeutic concentrations. To circumvent this challenge, a novel analog of curcumin, called C66, has been developed which requires only 5 mg/kg dose and it has been previously shown to alleviate diabetic cardiomyopathy and DN through in vitro and in vivo analyses [76,77]. Later, it was found that C66 inhibits miR-21 which plays a role in its protective mechanism against DN [78].

### 5.3. Small Molecules

Atorvastatin is a synthetic lipid-lowering drug used in the prevention and treatment of atherosclerosis and cardiovascular disease. It has been shown to reduce the miR-21 level [79]. In a study on the effects of atorvastatin in type 1 diabetes mellitus mice, it reduced the level of miR-21 that resulted in alleviating renal tubular epithelial cell injury. The authors first treated the streptozotocin-induced diabetic mice with high glucose and high fat, which increased the level of miR-21 and downregulated the expression of peroxisome proliferator-activated receptors-α (PPAR-α). Upon the administration of atorvastatin, these changes were reversed which led to an improvement in lipid metabolism, mitochondrial dysfunction, and subsequent reduction in DKD [80].

Metformin is another small molecule with the potential to downregulate miR-21. Metformin is better known for its hypoglycemic effects in T2DM but recently it was shown to have direct implications on DN by modulating miR-21 levels. First, it was found that T2DM and DN patients have an elevated level of miR-21-5p and a lower level of MMP-9 [81]. A similar analysis of patients receiving metformin revealed that they have a lower level of miR-21 and higher levels of MMP-9. The same study also found that metformin directly targets miR-21. Moreover, the in silico analysis showed that MMP-9 and PTEN are targets of miR-21-5p.

Pioglitazone is a PPAR-γ agonist widely used in T2DM. It possesses glucose lowering as well as direct anti-fibrotic properties and was shown to alleviate DN [82,83]. Recently, it was found that pioglitazone inhibits miR-21-5p expression in TGF-β1-exposed HK-2 cells and unilateral ureteral obstruction (UUO) kidney [84]. The inhibition of miR-21-5p expression by pioglitazone was confirmed when the administration of miR-21-5p inhibitors produced the same effect as pioglitazone and that of miR-21-5p mimics which reversed these effects [84].

### 5.4. Genetic Engineering

Although less practical than the above-mentioned intervention, genetic engineering provides another therapeutic option to lower the levels of miR-21. In an animal study, the researchers used ultrasound-mediated gene transfer to introduce a plasmid containing a knockdown sequence for miR-21 into the kidney cells of diabetic mice [21]. This plasmid was mixed with another plasmid to induce its expression. This knockdown led to the mitigation of microalbuminuria and renal fibrosis and inflammation in diabetic mice.

## 6. Future Possibilities in miRNA-Based Therapy

Current therapeutic interventions for DN targeting miR-21 are in the preclinical stages, with Lademirsen being the only relevant intervention that entered clinical studies for the treatment of Alport syndrome. However, the phase 2 clinical trial was eventually terminated due to failure to meet the objectives. Prior to clinical trials, any potential therapeutic must address a number of issues, along with safe and effective delivery to the site of action, the kidneys. The most promising agents to silence miR-21 are ASOs, which require subcutaneous or intravenous administration due to their low stability and poor absorption from the gastrointestinal tract. This poses potential complications for human subjects, and early clinical trials must address these concerns. Additionally, sustainable intervention with an extended half-life is necessary to avoid frequent injections and improve patient compliance. Despite these challenges, given that DN is a significant complication of diabetes, addressing these issues and pursuing clinical studies of miR-21 targeting interventions is a worthy endeavor.

## 7. Conclusions

Although current therapeutic approaches for DN-directing miR-21 are in preclinical stages, relevant studies have shown a significant potential for this miRNA as both diagnostics and therapeutic targets for renal pathologies. Emerging evidence has demonstrated that miR-21 is a pathogenic factor in the development of DN and its expression is dramatically enhanced in the course of DN. Consolidating and translating these findings, however, will take much more work. To integrate the therapeutic role of miR-21 into standard clinical practice and to confirm its viability, multination and multicenter epidemiological studies are needed. Nonetheless, the discovery of miRNAs as potential targets for the enhancement of DN patient outcomes offers optimism for important upcoming clinical advancements.

## Figures and Tables

**Table 1:** Therapeutic implications of miR-21 silencing in diabetic nephropathy.

Model	miR-21 Expression	Target	Major Findings	Ref.
Human and mouse	Upregulated	CDC25A, CDK6	miR-21 targets CDC25A and CDK6 in mesangial cells and results in mesangial cell hypertrophy by stimulating a G1-phase arrest. miR-21 targets PTEN, increasing podocyte motility as well as the formation and deposition of extracellular matrix.	[23]
MES13 cell line, mouse kidney, and human biopsies	Upregulated	TIMP3	A considerable overexpression of miR-21 was seen in mesangial cells cultured in high glucose environments and in mouse kidney and human kidney biopsies. The glycemic burden can stimulate miR-21 expression and destroy TIMP3 mRNA.	[54]
Diabetic kk-ay mice and C57BL mice (control)	Upregulated	MMP-9/TIMP1	miR-21 expression was significantly higher in kk-ay mice. miR-21 expression positively correlated with TIMP1, collagen IV, urine albumin creatine ratio (ACR), and fibronectin; whereas negatively correlated with creatine clearance ratio (Ccr) and MMP-9 protein. Antagomir-21 improved Ccr and ACR and reduced collagen IV, TIMP1, and fibronectin.	[55]
Male kk-ay and C57BL/6J mice	Upregulated	SMAD7	miR-21 overexpression accelerated TGF-β1-induced EMT by targeting SMAD7. Notably, miR-21 inhibitor improves the renal structure and function and inhibits fibrosis.	[31]
DN mouse models and cell models	Upregulated	FOXO1	FOXO1 was recognized as a target of miR-21. By specifically targeting FOXO1 in high glucose cultured podocytes, miR-21 utilizes its pro-apoptosis and anti-autophagy effects.	[47]
Rat renal tubular epithelial cells and HEK 293T cells	Upregulated	SMAD7	SMAD7 is a direct target of miR-21, and its overexpression may prevent rat renal tubular epithelial cells from proliferating.	[56]
db/db mice (a mouse model of T2D)	Upregulated	SMAD7	Overexpression of miR-21 in kidney cells increased the generation of fibrotic and inflammatory markers driven by high glucose, whereas miR-21 knockdown decreased this production. Renal miR-21 knockdown restored Smad7 levels and reduced activation of the TGF-β and NF-κB signaling pathways.	[21]
OVE26 type 1 diabetic mouse	Upregulated	PTEN, PRAS40	Upregulation of miR-21 resulted in the promotion of renal fibrosis. In high glucose-induced TORC1 activity, miR-21 increased renal cell hypertrophy and fibronectin expression.	[57]
Kidney biopsies of DN patients and normal kidney donors	Upregulated	PTEN-SMAD7	Tubular miR-21 upregulation was seen in human kidney biopsies. miR-21 specifically targets the repressors of SMAD3-dependent and PI3K-dependent TGF-β1 signaling, SMAD7, and PTEN (known fibrotic signaling proteins), respectively.	[29]
DN patients	Upregulated	Not determined	Patients with DN had higher levels of hsa-miR-21-5p, and an inverse relationship between eGFR and miR-21-5p in the proximal tubules and glomeruli was found.	[58]

Abbreviations: SMAD: mothers against decapentaplegic homolog; PTEN: phosphatase and TENsin homolog; DN: diabetic nephropathy; PI3K: phosphoinositide 3-kinase; PRAS40: proline-rich Akt substrate of 40 kDa; TGF-β: transforming growth factor-β; NF-κB: nuclear factor κB; TIMP: tissue inhibitor of metalloproteinases; MMP-9: matrix metalloproteinases-9; FOXO1: forkhead box O1; TORC1: target of rapamycin complex 1; eGFR: estimated glomerular filtration rate; T2D: Type 2 diabetes; CDC25A: cell division cycle 25A; CDK6: cyclin-dependent kinase 6.

## Data Availability

Not applicable.
